# Maternal risk associated with the VACTERL association: A case–control study

**DOI:** 10.1002/bdr2.1773

**Published:** 2020-07-22

**Authors:** Romy van de Putte, Hermien E. K. de Walle, Kirsten J. M. van Hooijdonk, Ivo de Blaauw, Carlo L. M. Marcelis, Arno van Heijst, Jacques C. Giltay, Kirsten Y. Renkema, Paul M. A. Broens, Erwin Brosens, Cornelius E. J. Sloots, Jorieke E. H. Bergman, Nel Roeleveld, Iris A. L. M. van Rooij

**Affiliations:** ^1^ Department for Health Evidence, Radboud Institute for Health Sciences Radboud university medical center (Radboudumc) Nijmegen the Netherlands; ^2^ Department of Genetics, University Medical Center Groningen University of Groningen Groningen the Netherlands; ^3^ Department for Pediatric Surgery Radboudumc Amalia Children's Hospital Nijmegen the Netherlands; ^4^ Department of Human Genetics, Radboud Institute for Molecular Life Sciences Radboudumc Nijmegen the Netherlands; ^5^ Department of Pediatrics – Neonatology Radboudumc Amalia Children's Hospital Nijmegen the Netherlands; ^6^ Division Laboratories, Pharmacy and Biomedical Genetics University Medical Center Utrecht Utrecht the Netherlands; ^7^ Department of Genetics, Center for Molecular Medicine, University Medical Center Utrecht Utrecht University Utrecht the Netherlands; ^8^ Department of Surgery, Division of Pediatric Surgery, University Medical Center Groningen University of Groningen Groningen the Netherlands; ^9^ Department of Clinical Genetics Erasmus Medical Centre Rotterdam the Netherlands; ^10^ Department of Pediatric Surgery Erasmus Medical Centre Sophia Children's Hospital Rotterdam the Netherlands

**Keywords:** assisted reproductive techniques, folic acid supplement use, overweight, parity, smoking

## Abstract

**Background:**

The VACTERL association (VACTERL) includes at least three of these congenital anomalies: vertebral, anal, cardiac, trachea‐esophageal, renal, and limb anomalies. Assisted reproductive techniques (ART), pregestational diabetes mellitus, and chronic lower obstructive pulmonary disorders (CLOPD) have been associated with VACTERL. We aimed to replicate these findings and were interested in additional maternal risk factors.

**Methods:**

A case–control study using self‐administered questionnaires was performed including 142 VACTERL cases and 2,135 population‐based healthy controls. Multivariable logistic regression analyses were performed to estimate confounder adjusted odds ratios (aOR) and 95% confidence intervals (95%CI).

**Results:**

Parents who used invasive ART had an increased risk of VACTERL in offspring (aOR 4.4 [95%CI 2.1–8.8]), whereas the increased risk for mothers with CLOPD could not be replicated. None of the case mothers had pregestational diabetes mellitus. Primiparity (1.5 [1.1–2.1]) and maternal pregestational overweight and obesity (1.8 [1.2–2.8] and 1.8 [1.0–3.4]) were associated with VACTERL. Consistent folic acid supplement use during the advised periconceptional period may reduce the risk of VACTERL (0.5 [0.3–1.0]). Maternal smoking resulted in an almost twofold increased risk of VACTERL.

**Conclusion:**

We identified invasive ART, primiparity, pregestational overweight and obesity, lack of folic acid supplement use, and smoking as risk factors for VACTERL.

## 1 INTRODUCTION

The VACTERL association consists of the following congenital anomalies: vertebral, anal, cardiac, tracheo‐esophageal, renal, and limb anomalies (Solomon, 2011; Temtamy & Miller, 1974). Patients are usually diagnosed when displaying three or more of these congenital anomalies (Solomon et al., 2012). In the majority of patients, VACTERL occurs sporadically, but an increased prevalence of component features in first‐degree relatives is observed (Hilger et al., 2012; Reutter & Ludwig, 2013; Salinas‐Torres, Perez‐Garcia, & Perez‐Garcia, 2015; Solomon, Pineda‐Alvarez, Raam, & Cummings, 2010). The prevalence of VACTERL was 9 in 100,000 births in the northern Netherlands in 1981–2015 (van de Putte et al., 2019). Although surgical techniques have improved over the last decades, VACTERL patients still experience problems related to their congenital anomalies, such as back pain related to vertebral anomalies or incontinence related to an anal anomaly/anorectal malformation (ARM; Raam, Pineda‐Alvarez, Hadley, & Solomon, 2011; Wheeler & Weaver, 2005).

Recently, several maternal risk factors for VACTERL in offspring were identified in a large European study: assisted reproductive techniques (ART), pregestational diabetes mellitus, and chronic lower obstructive pulmonary diseases (CLOPD; Van de Putte, 2019). Primiparity, previous miscarriages, and lifestyle factors, such as pregestational body mass index (BMI), folic acid supplement use, alcohol use, and smoking habits, were not included in that study, as information on these factors is not registered in a standardized manner across EUROCAT registries.

However, primiparity seemed to be associated with VACTERL (Czeizel & Ludanyi, 1985), as well as with isolated VACTERL component features (Duong et al., 2012; Oddsberg, Jia, Nilsson, Ye, & Lagergren, 2008; Wijers et al., 2013) in other studies. Previous miscarriages have been associated with ARM in offspring (van de Putte et al., 2017), while maternal overweight, alcohol use, and smoking may increase the risk of several congenital anomalies in offspring (Nicoletti, Appel, Siedersberger Neto, Guimaraes, & Zhang, 2014; Persson et al., 2017; Taruscio et al., 2014). Folic acid supplement use is recommended before and during pregnancy as it is known to reduce neural tube defects (Czeizel, 2009; Milunsky et al., 1989; MRC Multivitamin Study Research Group, 1991). Moreover, folic acid supplement use seems to reduce the risks of specific congenital anomalies such as anorectal, cardiac, trachea‐esophageal, limb, and urinary tract anomalies (Botto, Olney, & Erickson, 2004; Czeizel, 1998, 2009; Feng, Chen, Li, & Mo, 2016). Whether primiparity, previous miscarriages, and the above‐mentioned lifestyle factors are involved in the etiology of VACTERL remains largely unknown as these risk factors were only studied among small study populations or among patients with only one VACTERL component feature (Czeizel & Ludanyi, 1985; van Rooij et al., 2010). Therefore, the aim of this study was to replicate the findings of the previous European study on maternal risk factors for VACTERL (Van de Putte, 2019) in a Dutch study population and to investigate additional maternal risk factors associated with VACTERL in offspring.

## 2 METHODS

### 2.1 Study population

We included VACTERL cases (birth years 1985–2018) and healthy controls (birth years 1990–2011) from the Dutch AGORA (Aetiologic research into Genetic and Occupational/environmental Risk factors for Anomalies in Children) data‐ and biobank, initiated at the Radboud university medical center (Radboudumc). The AGORA data‐ and biobank contain clinical data, questionnaire data, and DNA samples from children with congenital anomalies or childhood cancers, population‐based healthy controls, and their parents (van Rooij et al., 2016). Parents of children with congenital anomalies are invited to participate in AGORA at the child's first consultation in the Radboudumc. When parents agree to participate, they are asked to complete a questionnaire that includes questions on demographics, family history, and health and lifestyle in the 3 months before and during pregnancy. The AGORA data and biobank expanded their inclusion of cases toward the clinical genetics department of the University Medical Center Utrecht (UMCU) and the pediatric surgery departments of the University Medical Center Groningen (UMCG) and the Erasmus Medical Center (Erasmus MC) in Rotterdam. The population‐based healthy controls, obtained through municipalities throughout the Netherlands, and cases that were counseled at the additional hospitals, were retrospectively invited for participation by regular mail.

In addition, we included VACTERL cases (birth years 1985–2018) from the EUROCAT registry that covers the northern part of the Netherlands (EUROCAT‐NNL). When a case was both present in AGORA and EUROCAT‐NNL, the data were provided from AGORA only to prevent duplicate cases (n = 5). Approximately half of the EUROCAT‐NNL cases (n = 29) were also included in a previous large European study on maternal risk factors for VACTERL in offspring (Van de Putte, 2019). EUROCAT is a European registry network that routinely collects standardized data on congenital anomalies throughout Europe (Kinsner‐Ovaskainen et al., 2018). Data in EUROCAT‐NNL are ascertained by registry personnel via multiple data sources, such as hospital records, obstetric records, records from the clinical genetics department, and post mortem examinations. From 1997 onward, EUROCAT‐NNL has collected parental questionnaire data from children with congenital anomalies as well. These questionnaires include questions comparable to those in the AGORA questionnaires.

The Regional committee on Research Involving Human Subjects Arnhem‐Nijmegen approved the data collection protocol of the AGORA data and biobank and all participants and/or their parents gave written informed consent. Parents of cases from EUROCAT‐NNL gave written informed consent for their data to be registered and used for research on congenital anomalies. As anonymous data were obtained from EUROCAT‐NNL, additional ethical approval was not required for sharing of the data.

### 2.2 Case and control definitions

From AGORA, we selected 103 VACTERL cases (born ≥1985) with available maternal questionnaire data, diagnosed in the clinic by a clinical geneticist, a pediatric surgeon, or neonatologist. From EUROCAT‐NNL, we selected 63 additional VACTERL cases, selected based on ICD‐9‐BPA codes 759895 and 75989, ICD‐10‐BPA code Q8726, or OMIM/McKusick codes 192350, 314390, and 276950. Cases with an ICD‐9‐BPA code 75989 were only selected when VATER/VACTERL was specified in the text, as this code is not specific for VACTERL. These cases included live births, stillbirths (gestational age 24 weeks or more), miscarriages (gestational age less than 24 weeks), and terminations of pregnancy for fetal anomaly following prenatal diagnosis (TOPFA). We excluded cases with VACTERL and hydrocephalus (n = 7), as this is considered a different condition with a suggested autosomal recessive or X‐linked inheritance (OMIM **#**314390). Cases with a syndrome that explained their phenotype, including caudal regression syndrome, and Townes‐Brocks syndrome were excluded as well (n = 5).

The recently introduced VACTERL limits were used to discriminate between major and minor VACTERL features, and additional major and minor congenital anomalies (van de Putte et al., 2019). In addition, these guidelines were used to classify our cases in three mutually exclusive subtypes: STRICT‐VACTERL, VACTERL‐LIKE, and VACTERL‐PLUS, and to exclude the subgroup of NO‐VACTERL cases (n = 12), consisting of cases originally diagnosed with VACTERL but actually not fully complying with the diagnostic criteria (van de Putte et al., 2019). This left us with 142 true VACTERL cases.

The population‐based control group consisted of healthy children that were born in 1990–2011 and were randomly sampled in 2010–2011 via 39 municipalities in geographical areas comparable to those of the AGORA cases. We excluded 14 controls without maternal questionnaire data and 59 controls with major congenital anomalies, a known genetic syndrome, or a chromosomal abnormality based on the answers given by the parents in the questionnaires, leaving 2,135 controls.

### 2.3 Determinant definitions

The infant characteristics included were sex, birth year, birth type (live birth, miscarriage, stillbirth, and TOPFA), birth weight (in grams), and gestational age (in completed weeks). Maternal characteristics taken into account were age at childbirth (in years), education (low: no, primary, lower vocational, or intermediate secondary education; middle: intermediate vocational or higher secondary education; and high: higher vocational or academic education), and medical factors such as primiparity (vs. multiparity), multiple pregnancy (vs. singleton pregnancy), previous miscarriages, the use of ART (including in vitro fertilization [IVF], intracytoplasmic sperm injection [ICSI], gamete intrafallopian transfer [GIFT], egg donation, artificial insemination, and induced ovulation), chronic illnesses (pregestational diabetes mellitus and CLOPD), and medication taken before or during pregnancy. In addition, the following maternal lifestyle factors were included: pregestational body mass index (BMI), folic acid supplement use, alcohol use, and smoking. Paternal characteristics included were age at childbirth (in years), education, and smoking.

Maternal and paternal age at childbirth were categorized into <20, 20–34, and ≥35 years. For ART, we distinguished between invasive and noninvasive techniques with IVF, ICSI, GIFT, and egg donation being considered invasive ARTs, whereas artificial insemination and induced ovulation were considered noninvasive, as these procedures did not involve gamete manipulation (Davies et al., 2012). For pregestational diabetes mellitus, the following codes were included: ICD‐10 codes E10‐E14, P701, O240, and O241; and ICD‐9 codes 2500‐2509, 7750, and 6480. For CLOPD, the ICD‐10 codes were J40‐J47 and the ICD‐9 codes 490‐496. BMI was categorized into four groups: underweight <18.5 kg m^2^, normal 18.5–24.9 kg m^2^, overweight 25.0–29.9 kg m^2^, and obese ≥30 kg m^2^.

In the Netherlands, women are advised to use 400 μg folic acid starting 4 weeks before conception through 8 weeks after conception (de Walle & de Jong‐van den Berg, 2008). We excluded cases and controls born before 1995 from the analyses on folic acid supplement use, because the advice to use folic acid supplements in the periconceptional period was first published in November 1993. Folic acid supplement use was split into two, not mutually exclusive, time periods: (a) supplement use during the advised period and (b) supplement use during the etiologically relevant period for the VACTERL association, defined as intake from Weeks 3 through 10 after conception in which the organ systems related to VACTERL develop. For each time period, we further divided the women into (a) partial users, defined as women who used folic acid supplements at any point during the time period, and (b) consistent users, defined as women who consistently used folic acid supplements during the entire time period. Nonusers were defined as women who reported not to have used folic acid supplements at all and women who started using folic acid supplements after the advised period or from Week 11 after conception onward, respectively. When information on the time of usage was missing or unclear, women were excluded from the analyses. Both folic acid supplements used as a single tablet and folic‐acid containing multivitamins especially for pregnant women were included as folic acid supplements. We excluded regular multivitamins (1.5 and 0.9% of women in the advised and etiologically relevant period), as they generally did not contain the advised dose of 400 μg folic acid.

Similar to the above, mothers were also divided into partial, consistent, and nonusers for alcohol use and smoking habits during the etiologically relevant period for the VACTERL association (weeks 3 through 10 after conception). Smoking of the father was not divided into partial, consistent, or nonusers as this level of detail was not available. Paternal smoking during the first 4 months of pregnancy was used as a proxy for passive maternal smoking.

### 2.4 Statistical analyses

We used SPSS 25.0 (SPSS Inc., Chicago, IL) for the statistical analyses. Crude and adjusted odds ratios (OR and aOR) with 95% confidence intervals (CIs) were estimated using univariable and multivariable logistic regression analyses. We selected birth year, maternal age at childbirth, and time between childbirth and completion of the maternal questionnaire as potential confounders, as well as all determinants that were not the primary factor of interest in the specific analyses, including primiparity, multiple pregnancy (for analyses of lifestyle factors only), previous miscarriages, ART (stratified into invasive and noninvasive), pregestational diabetes mellitus, CLOPD, pregestational BMI, folic acid supplement use, alcohol use, and maternal smoking. All potential confounders were included in the full model for each determinant, from which variables were excluded when the OR did not change >10% upon removal.

The following sensitivity analyses were performed: (a) by excluding all cases born after 2011, as no controls were available from this period; (b) by expanding the groups of mothers with chronic illnesses with women having relevant medication use in the first 10 weeks after conception (anatomical therapeutic chemical [ATC] codes: A10 for medication used in diabetes mellitus and R03 for medication used for obstructive airway diseases); (c) by including mothers who did not specify the exact period of folic acid supplement use as consistent users, as partial users, and as nonusers in three consecutive simulated analyses for folic acid supplement use.

## 3 RESULTS

The final study population consisted of 142 VACTERL cases and 2,135 healthy controls. The VACTERL cases were categorized into 60 (42%) STRICT‐VACTERL cases, 51 (36%) VACTERL‐LIKE cases, and 31 (22%) VACTERL‐PLUS cases. Table 1 shows the percentage of the major VACTERL component features among the cases as specified previously (van de Putte et al., 2019). Anal anomalies were seen in most cases (72%), followed by cardiac (53%) and trachea‐esophageal (51%) anomalies. In Table 2, further characteristics of our study population are described. The majority of the cases were male (64%), whereas the percentages of male and female controls were equal. The majority of cases were live born (92%), whereas the controls only included live born children. The cases more often had a low birth weight or were born preterm compared to the controls (42 vs. 7% and 37 vs. 9%, respectively). Parental age at childbirth and parental education were similar for the case and control groups. The median age of the children at the time of completing the questionnaire was lower in cases (4 years, range 0–30 years) than in controls (10 years, range 0–21 years).

**TABLE 1:** The numbers and percentages of major VACTERL component features present in the 142 VACTERL cases included

	*N*	%
Vertebral anomalies	56	39.4
Anal anomalies	102	71.8
Cardiac anomalies	75	52.8
Tracheo‐esophageal anomalies	72	50.7
Renal anomalies	60	42.3
Limb anomalies	27	19.0

*Note:* See van de Putte et al., 2019 for the specification of the congenital anomalies that belong to the major VACTERL component features.

**TABLE 2:** Infant, maternal, and paternal characteristics of VACTERL cases and controls

	Cases (*N* = 142)^a^ *N* (%)	Controls (*N* = 2,135)^a^ *N* (%)
Sex		
Male	91 (64.1)	1,048 (49.1)
Female	51 (35.9)	1,087 (50.9)
Year of birth		
<1997	39 (27.5)	627 (29.4)
1997–2000	26 (18.3)	425 (19.9)
2001–2005	24 (16.9)	502 (23.5)
2006–2010	25 (17.6)	577 (27.0)
2011–2018	28 (19.7)	4 (0.2)
Birth type		
Live birth	130 (91.5)	2,135 (100.0)
Stillbirth	6 (4.2)	
TOPFA	5 (3.5)	
Miscarriage	1 (0.7)	
Low birth weight (<2,500 g)^b^	51 (42.1)	144 (6.9)
Preterm birth (<37 weeks)^b^	46 (37.1)	185 (8.8)
Maternal age at childbirth		
<20 years		7 (0.3)
20–34 years	110 (77.5)	1,648 (77.6)
≥35 years	32 (22.5)	470 (22.1)
Paternal age at childbirth		
<20 years	1 (0.8)	
20–34 years	75 (57.7)	1,085 (57.2)
≥35 years	54 (41.5)	812 (42.8)
Maternal education		
Low	21 (17.8)	369 (17.3)
Middle	55 (46.6)	985 (46.2)
High	42 (35.6)	777 (36.5)
Paternal education		
Low	23 (21.1)	418 (22.0)
Middle	42 (38.5)	748 (39.3)
High	44 (40.4)	735 (38.7)
Age child at completion of maternal questionnaire in years, median (range)	4 (0–30)	10 (0–21)

Abbreviations: TOPFA, terminations of pregnancy for fetal anomaly following prenatal diagnosis.

^a^ Numbers do not add up to total number due to missing values, which were below 2.0% for all variables except low birth weight (2.7%), paternal age at childbirth (11.%), paternal education (11.7%), and age child at completion of maternal questionnaire (4.6%).

^b^ Only calculated for live births.

The associations between the potential medical and lifestyle risk factors and VACTERL in offspring are shown in Tables 3 and 4. We observed an association between primiparity and VACTERL (aOR 1.5 [95%CI 1.1–2.1]). Couples who conceived through ART also had an increased risk of having a child with VACTERL (aOR 2.4 [95%CI 1.4–4.2]). However, the risk was only truly increased among couples who used invasive ART (aOR 4.4 [95%CI 2.1–8.8]). When we stratified for specific invasive techniques, we observed different risk estimates for couples who used IVF and ICSI (aOR 5.2 [95%CI 2.0–13.5] and aOR 2.7 [95%CI 0.9–8.0], respectively). Contrary to expectation, the OR for offspring of mothers with CLOPD was not clearly increased (aOR 1.7 [95%CI 0.7–3.8]), while none of the case mothers had pregestational diabetes mellitus. Multiple pregnancy (aOR 1.5 [95%CI 0.7–3.1] when ART was included in the model) and previous miscarriages (OR 1.3 [95%CI 0.9–2.0]) did not seem to be associated with VACTERL in offspring.

**TABLE 3:** Associations between maternal medical factors and the VACTERL association in offspring

	Total cases/controls^a^ (*N* = 142/2,135)	Missing data (%)	Cases and controls with potential risk factor, *N* (%)		Crude OR (95% CI)	Adjusted OR (95% CI)
Primiparity	140/2,128	0.4	78 (55.7)	968 (45.5)	1.5 (1.1–2.1)	1.5 (1.1–2.1)^b^
Multiple pregnancy	129/2,128	0.9	10 (7.8)	80 (3.8)	2.2 (1.1–4.3)	1.5 (0.7–3.1)^c^
Previous miscarriages	133/2,122	1.0	31 (23.3)	396 (18.7)	1.3 (0.9–2.0)	1.3 (0.9–2.0)^b^
ART	114/2,051	5.0	16 (14.0)	120 (5.9)	2.6 (1.5–4.6)	2.4 (1.4–4.2)^d^
Noninvasive	114/2,050	5.0	5 (4.4)	74 (3.6)	1.3 (0.5–3.4)	1.2 (0.5–3.1)^d^
Invasive	114/2,050	5.0	11 (9.6)	45 (2.2)	4.8 (2.4–9.6)	4.4 (2.1–8.8)^d^
Pregestational diabetes	121/2,126	1.3	0 (0.0)	7 (0.3)		
CLOPD	115/1,994	7.4	7 (6.1)	57 (2.9)	2.2 (1.0–4.9)	1.7 (0.7–3.8)^e^

Abbreviations: ART, assisted reproductive techniques; CI, confidence interval; CLOPD, chronic lower obstructive pulmonary disorders; OR, odds ratio.

*Note:* For ART, we distinguished between invasive and noninvasive techniques with IVF, ICSI, GIFT, and egg donation being considered invasive ARTs, whereas artificial insemination and induced ovulation were considered noninvasive, as these procedures did not involve gamete manipulation. ORs were estimated if ≥3 cases were exposed.

^a^ Some cases and controls were excluded in the specific analysis because of missing data on the determinant and/or the confounder(s).

^b^ None of the potential confounders proved to be true confounders.

^c^ Adjusted for ART (three categories).

^d^ Adjusted for age child at completion of maternal questionnaire (in years).

^e^ Adjusted for the pregestational BMI and birth year categories.

**TABLE 4:** Associations between maternal lifestyle factors and the VACTERL association in offspring

	Total cases/controls^a^ (*N* = 142/2,135)	Missing data (%)	Cases and controls with potential risk factor, *N* (%)		Crude OR (95%CI)	Adjusted OR (95%CI)
Maternal pregestational BMI	120/1,996	7.1				
Underweight			5 (4.2)	69 (3.5)	1.5 (0.6–3.9)	1.7 (0.7–4.4)^f^
Normal			69 (57.5)	1,433 (71.8)	Reference	Reference
Overweight			33 (27.5)	364 (18.2)	1.9 (1.2–2.9)	1.8 (1.2–2.8)^f^
Obesity			13 (10.8)	130 (6.5)	2.1 (1.1–3.9)	1.8 (1.0–3.4)^f^
Maternal folic acid supplement use^b^						
Advised period^c^	86/1,289	24.8				
Partial use			36 (41.9)	490 (38.0)	1.2 (0.7–2.0)	0.7 (0.4–1.3)^f^
Consistent use			27 (31.4)	429 (33.3)	1.0 (0.6–1.8)	0.5 (0.3–1.0)^f^
Etiologically relevant period^d^	91/1,403	18.3				
Partial use			20 (22.0)	251 (17.9)	1.4 (0.8–2.7)	0.9 (0.5–1.6)^f^
Consistent use			46 (50.5)	699 (49.8)	1.2 (0.7–2.0)	0.6 (0.4–1.1)^f^
Maternal alcohol use^e^	121/2,094	2.7				
Partial use			11 (9.1)	170 (8.1)	1.1 (0.6–2.1)	1.1 (0.6–2.0)^f^
Consistent use			7 (5.8)	126 (6.0)	1.0 (0.4–2.1)	1.1 (0.5–2.5)^f^
Maternal smoking^e^	126/2,125	1.1				
Partial use			2 (1.6)	71 (3.3)		
Consistent use			25 (19.8)	298 (14.0)	1.5 (0.9–2.3)	1.9 (1.2–3.0)^f^
Paternal smoking^e^	121/1,874	12.4	38 (31.4)	540 (28.8)	1.1 (0.8–1.7)	1.1 (0.8–1.7)^g^

Abbreviations: BMI, body mass index.

*Note:* Partial use was defined as folic acid supplement use, alcohol use, or smoking at any point during the time period. Consistent use was defined as folic acid supplement use, alcohol use, or smoking during the entire time period.

^a^ Some cases and controls were excluded in the specific analysis because of missing data on the determinant and/or the confounder(s).

^b^ Cases and controls born after 1994 were included only (*N* = 112/1,716 in total).

^c^ Advised period: 4 weeks before pregnancy through Week 8 after conception.

^d^ Etiologically relevant period: Week 3 through Week 10 after conception.

^e^ During the etiologically relevant period: Week 3 through Week 10 after conception.

^f^ Adjusted for the birth year categories.

^g^ None of the potential confounders proved to be true confounders.

In Table 4, we observed increased risks of having a child with VACTERL among mothers with overweight (aOR 1.8 [95%CI 1.2–2.8]) and obesity (aOR 1.8 [95%CI 1.0–3.4]), compared to mothers with a normal BMI. Partial use of folic acid supplements was not associated with reduced risks of VACTERL in offspring (aOR 0.7 [95%CI 0.4–1.3] and aOR 0.9 [95%CI 0.5–1.6] for advised and etiologically relevant period, respectively). Consistent folic acid supplement use, however, may reduce the risk of VACTERL in offspring in both periods (aOR 0.5 [95%CI 0.3–1.0] and aOR 0.6 [95%CI 0.4–1.1]). Maternal smoking during the entire etiologically relevant period doubled the risk of VACTERL in offspring. As only two case mothers smoked partially during the etiologically relevant period, no OR could be estimated. Paternal smoking during pregnancy was not associated with VACTERL in offspring and neither was maternal alcohol use.

In the sensitivity analyses without cases born after 2011, 25 cases were excluded. Similar risk estimates were obtained as presented in Tables 3 and 4, except for slightly higher risks for multiple pregnancy (aOR 2.0 [95%CI 1.0–4.2]), CLOPD (aOR 1.9 [95%CI 0.8–4.6]), and overweight and obesity (aOR 2.4 [95%CI 1.5–3.8] and aOR 2.1 [95%CI 1.1–4.3], respectively), and attenuation of the risk reduction by consistent folic acid supplement use in both advised and etiologically relevant periods (aOR 0.6 [95%CI 0.3–1.2] and aOR 0.7 [95%CI 0.4–1.2]; Tables S1 and S2, Supporting Information). A small percentage of mothers were identified as having a chronic illness based on medication use only. When these mothers were included in the analyses, the aOR for CLOPD increased to 2.1 [95%CI 1.0–4.4]; Table S3). In the simulation analyses including mothers who did not specify the exact period of folic acid supplement use as consistent, partial, or nonusers, we observed similar risk estimates when mothers were assumed to be partial or consistent users, but the beneficial effects of folic acid supplement use disappeared when these mothers were assumed to be nonusers (Table S4).

## 4 DISCUSSION

In this case–control study, we replicated the previous finding of ART as a risk factor for the VACTERL association in offspring. In addition, we observed that invasive ART only seems to be a risk factor for VACTERL in offspring. Furthermore, we identified primiparity, pregestational overweight and obesity, consistent lack of folic acid supplement use, and consistent smoking during the etiologically relevant period (Weeks 3 through 10 after conception) as maternal risk factors for VACTERL. We did not observe associations with CLOPD, pregestational diabetes mellitus, multiple pregnancy, previous miscarriages, partial folic acid supplement use, alcohol use, and paternal smoking.

An important strength of this study is the inclusion of a healthy population‐based control group as opposed to a control group with congenital anomalies that may also be caused by risk factors of interest to VACTERL, such as smoking or ART. We performed the largest case–control study with healthy controls to date, with a case population that was quite similar in size compared to the recent European case–malformed control study in which maternal risk factors were identified (Van de Putte, 2019). The actual case population of the European study was larger (n = 329), but the number of cases that were included in the specific analyses were comparable to our study for most risk factors. Unfortunately, the number of exposed cases was still too small to perform stratified analyses for the previously proposed VACTERL subtypes.

Another strength is the addition of a number of sensitivity analyses, in many of which similar risk estimates were observed as in the primary analysis, suggesting robust results. Some risk estimates were slightly higher or lower in the sensitivity analyses, but this did not change the conclusions of our study for the majority of factors studied. However, when we excluded cases born after 2011 for better comparability with the controls regarding birth year, we identified multiple pregnancy as an extra independent risk factor for having a child with VACTERL. The absolute number of twins was similar to the original analysis, but because of the smaller group of cases, the percentage of twins increased. This resulted in a different risk estimate when ART was included in the model, probably due to single embryo transfer being more common during ART procedures in the period after 2011. CLOPD proved to be an independent risk factor for VACTERL when we included medication use to identify mothers with a chronic illness. This may point to underestimation due to random misclassification in the primary analysis, if mothers with for example asthma did not report the disease because it was fully controlled by asthma medication. Random misclassification may also explain the disappearance of the beneficial effect of folic acid supplement use in the simulation analyses in which women who reported folic acid use without an exact time period of use were treated as nonusers.

A limitation of this study may be the difference in the time lag between childbirth and completing the maternal questionnaire for cases and controls, potentially leading to some misclassification. Primiparity and multiple pregnancy are not prone to this misclassification and other maternal factors under study, such as miscarriages, ART, and having diabetes mellitus, or CLOPD, are also likely to be remembered well, as they usually have a large impact. In contrast, a longer time lag between childbirth and completing the questionnaire may have resulted in more recall errors regarding the lifestyle factors studied. As small numbers prevented us from performing stratified analyses, we included the time lag as a potential confounder in all analyses. As it did not prove to be a true confounder, however, we expect differential misclassification due to the different time lag to be minimal.

The association between the use of ART and VACTERL in offspring in this study was in the same order of magnitude as in the recently published European study (aOR 2.4 [1.4–4.2] and aOR 2.3 [1.3–3.9], respectively; Van de Putte, 2019). As we were able to differentiate between invasive and noninvasive ART, we identified that only couples who used invasive ART were at risk, suggesting that gamete manipulation is involved in the etiology of VACTERL (Davies et al., 2012). This study may even suggest that couples who used IVF were more at risk compared to couples who used ICSI, but the numbers are too low to draw reliable conclusions. It is also possible that couples that used invasive ART have more complex fertility issues compared to couples who used noninvasive ART, indicating that the underlying subfertility may play a role.

We were not able to replicate the association between CLOPD and VACTERL in offspring in our primary analysis, but our crude risk estimate pointed in the same direction as previously identified in the European study (OR 2.2 [95%CI 1.0–4.9] and aOR 3.9 [95%CI 2.2–6.7], respectively (Van de Putte, 2019)). In addition, an aOR of 2.1 [95%CI 1.0–4.4] was seen in the sensitivity analysis when maternal medication use was also taken into account to identify mothers with a chronic illness. These findings strengthen the hypothesis that CLOPD is involved in the etiology of the VACTERL association. However, we cannot rule out an effect of residual confounding by BMI in the European study, as that study did not include information on BMI (Van de Putte, 2019). In the current study, information on pregestational BMI was available, and adjustment for the BMI and birth year categories attenuated our risk estimate to aOR 1.7 [95%CI 0.7–3.8]. The increased risk for pregestational diabetes mellitus observed in the European study could not be replicated or refuted due to a lack of case mothers with pregestational diabetes mellitus.

In other studies, primiparity was identified as a risk factor for VACTERL (Czeizel & Ludanyi, 1985), as well as for some of the VACTERL component features, including ARM, esophageal atresia with or without tracheo‐esophageal fistula (EA/TEF), cardiac, and limb anomalies (Duong et al., 2012; Oddsberg et al., 2008; Wijers et al., 2013). We were able to replicate the finding for the VACTERL association in the current study. This association may be explained by biological differences between primiparous pregnancies and multiparous pregnancies, such as a smaller and less vascularized uterus among primiparous pregnancies (Rovas, Sladkevicius, Strobel, & Valentin, 2006).

Mothers with overweight and obesity had a twofold increased risk of having a child with VACTERL. Overweight was previously identified as risk factor for some of the component features of VACTERL, including ARM, cardiac, renal, and limb anomalies (In 't Woud et al., 2016; Macumber, Schwartz, & Leca, 2017; Stothard, Tennant, Bell, & Rankin, 2009; Svenningsson, Gunnarsdottir, & Wester, 2018; Wijers et al., 2014; Zheng et al., 2018), but not for EA/TEF (Oddsberg et al., 2008; Stothard et al., 2009). Possible mechanisms through which maternal overweight could contribute to the etiology of VACTERL are nutritional deficiencies or undiagnosed diabetes mellitus or hyperglycemia (Stothard et al., 2009).

Consistent folic acid supplement use during the entire advised or etiologically relevant period may reduce the risk of VACTERL in offspring. Folic acid supplement use is recommended to reduce the risk of neural tube defects in offspring (Czeizel, 2009; Milunsky et al., 1989; MRC Multivitamin Study Research Group, 1991), while it also seems to reduce the risk of some of the isolated VACTERL components including ARM, cardiac, trachea‐esophageal, and limb anomalies (Botto et al., 2004; Czeizel, 1998, 2009; Feng et al., 2016). Therefore, this study adds evidence to support the recommendation to use folic acid supplements before and during pregnancy.

Furthermore, we identified consistent maternal smoking during the etiologically relevant period as a risk factor for VACTERL. An association between smoking and congenital cardiac anomalies was identified before (Zhang et al., 2017), whereas associations were not observed for ARM, EA/TEF, renal, and limb anomalies (In 't Woud et al., 2016; van Rooij et al., 2010; Wong‐Gibbons et al., 2008; Zhang et al., 2017). An important limitation of some of these studies is that they investigated any smoking, and did not differentiate between consistent and partial use (In 't Woud et al., 2016; van Rooij et al., 2010; Wong‐Gibbons et al., 2008). Therefore, it remains difficult to compare these study results. Based on our study and for other reasons, such as the fact that smoking is known to decrease birth weight (Ward, Lewis, & Coleman, 2007), we would strongly advise women to stop smoking before they want to conceive.

## 5 CONCLUSION

We identified invasive ART, primiparity, pregestational overweight and obesity, lack of folic acid supplement use, and smoking as maternal risk factors involved in the etiology of VACTERL. These risk factors are not unique for VACTERL, as they have also been reported in relation to VACTERL component features, indicating that the etiology of VACTERL may not be different from that of the component features. In conclusion, this study supports a multifactorial etiology for VACTERL, in which both medical and lifestyle factors seem to be involved in addition to genetic factors, providing leads for preventive measures.

## CONFLICT OF INTEREST

The authors declare no potential conflict of interest.

## Supporting information


**Table S1.** Associations between maternal medical factors and the VACTERL association in offspring when excluding the 25 cases born after 2011.
**Table S2.** Associations between maternal lifestyle factors and the VACTERL association in offspring when excluding the 25 cases born after 2011.
**Table S3.** Associations between maternal chronic illnesses and the VACTERL association in offspring when maternal medication use was also taken into account to identify mothers with a chronic illness.
**Table S4.** Associations between folic acid supplement use and the VACTERL association in offspring by including mothers who reported folic acid use, but did not specify the exact period of folic acid supplement use as (a) consistent users, (b) partial users, and (c) nonusers in three consecutive simulated analyses.

## Data Availability

The data that support the findings of this study are available from the EUROCAT Northern Netherlands registry and the AGORA data‐ and biobank. Restrictions apply to the availability of these data, which were used under license for this study. Data are available on request to JRC‐EUROCAT@ec.europa.eu and agora.hev@radboudumc.nl with the permission of the registry and data‐ and biobank.
