# Supplementary material for: Maternal risk associated with the VACTERL association: A case–control study
Source: Birth Defects Res. 2020 Jul 22;112(18):1495–504. doi: 10.1002/bdr2.1773 (PMC7689936; doi:10.1002/bdr2.1773)
Supplement: Supplementary file 1 — Table S1. Associations between maternal medical factors and the VACTERL association in offspring when excluding the 25 cases born after 2011. Table S2. Associations between maternal lifestyle factors and the VACTERL association in offspring when excluding the 25 cases born after 2011. Table S3. Associations between maternal chronic illnesses and the VACTERL association in offspring when maternal medication use was also taken into account to identify mothers with a chronic illness. Table S4. Associations between folic acid supplement use and the VACTERL association in offspring by including mothers who reported folic acid use, but did not specify the exact period of folic acid supplement use as (a) consistent users, (b) partial users, and (c) nonusers in three consecutive simulated analyses. [file BDR2-112-1495-s001.docx]

Supplemental Table 1a. Associations between maternal medical factors and the VACTERL association in offspring when excluding the 25 cases born after 2011.

|  | Total  cases/controls^a^  (N=117/2,135) | Missing  Data | Cases and controls with potential risk factor  N (%) | | Crude OR  (95% CI) | Adjusted OR  (95% CI) |
| --- | --- | --- | --- | --- | --- | --- |
| Primiparity | 115/2,128 | 0.4% | 66 (57.4) | 968 (45.5) | **1.6 (1.1-2.4)** | **1.6 (1.1-2.4)^b^** |
| Multiple pregnancy | 106/2,128 | 0.8% | 10 (9.4) | 80 (3.8) | **2.7 (1.3-5.3)** | **2.0 (1.0-4.2)^c^** |
| Previous miscarriages | 108/2,122 | 1.0% | 22 (20.4) | 396 (18.7) | 1.1 (0.7-1.8) | 1.3 (0.9-1.9)^b^ |
| ART | 93/2,051 | 4.8% | 12 (12.9) | 120 (5.9) | **2.4 (1.3-4.5)** | **2.3 (1.2-4.3**)**^d^** |
| Non-invasive | 93/2,050 | 4.8% | 4 (4.3) | 74 (3.6) | 1.3 (0.5-3.6) | 1.2 (0.4-3.4)^d^ |
| Invasive | 93/2,050 | 4.8% | 8 (8.6) | 45 (2.2) | **4.2 (1.9-9.3)** | **4.1 (1.8-8.9)^d^** |
| Pregestational diabetes | 99/2,126 | 1.2% | 0 (0.0) | 7 (0.3) | - | - |
| CLOPD | 94/1,994 | 7.3% | 6 (6.4) | 57 (2.9) | 2.3 (1.0-5.5) | 1.9 (0.8-4.6)^e^ |

ART = assisted reproductive techniques, CLOPD = chronic lower obstructive pulmonary disorders, OR = odds ratio, CI = confidence interval. For ART, we distinguished between invasive and non-invasive techniques with IVF, ICSI, GIFT, and egg donation being considered invasive ARTs, whereas artificial insemination, and induced ovulation were considered non-invasive, as these procedures did not involve gamete manipulation. ORs were estimated if ≥3 cases were exposed. ^a^ Some cases and controls were excluded in the specific analysis because of missing data on the determinant and/or the confounder(s). ^b^ None of the potential confounders proved to be true confounders. ^c^ Adjusted for ART (3 categories).  ^d^ Adjusted for age child at completion of maternal questionnaire (in years). ^e^ Adjusted for the pregestational BMI and birth year categories.

Supplemental Table 1b. Associations between maternal lifestyle factors and the VACTERL association in offspring when excluding the 25 cases born after 2011.

|  | Total  cases/controls  (N=117/2,135)^a^ | Missing  data | Cases and controls with  potential risk factor N (%) | | Crude OR  (95% CI) | Adjusted OR  (95% CI) |
| --- | --- | --- | --- | --- | --- | --- |
| Maternal pregestational BMI | 96/1,996 | 7.1% |  |  |  |  |
| Underweight |  |  | 4 (4.2) | 69 (3.5) | 1.6 (0.6-4.6) | 1.6 (0.6-4.7)^f^ |
| Normal |  |  | 51 (53.1) | 1,433 (71.8) | Reference | Reference |
| Overweight |  |  | 31 (32.3) | 364 (18.2) | **2.4 (1.5-3.8)** | **2.4 (1.5-3.8)^f^** |
| Obesity |  |  | 10 (10.4) | 130 (6.5) | **2.2 (1.1-4.4)** | **2.1 (1.1-4.3)**^f^ |
| Maternal folic acid supplement use^b^ |  |  |  |  |  |  |
| Advised period^c^ | 65/1,289 | 24.9% |  |  |  |  |
| Partial use |  |  | 24 (36.9) | 490 (38.0) | 0.8 (0.4-1.4) | 0.7 (0.4-1.3)^f^ |
| Consistent use |  |  | 18 (27.7) | 429 (33.3) | 0.7 (0.4-1.3) | 0.6 (0.3-1.2)^f^ |
| Etiologically relevant period^d^ | 69/1,403 | 18.4% |  |  |  |  |
| Partial use |  |  | 15 (21.7) | 251 (17.9) | 1.1 (0.6-2.1) | 1.0 (0.5-1.9)^f^ |
| Consistent use |  |  | 29 (42.0) | 699 (49.8) | 0.8 (0.4-1.3) | 0.7 (0.4-1.2)^f^ |
| Maternal alcohol use^e^ | 101/2,094 | 2.8% |  |  |  |  |
| Partial use |  |  | 8 (8.3) | 170 (8.1) | 1.0 (0.5-2.2) | 1.0 (0.5-2.2)^f^ |
| Consistent use |  |  | 7 (7.3) | 126 (6.0) | 1.2 (0.6-2.7) | 1.3 (0.6-2.8)^f^ |
| Maternal smoking^e^ | 101/2,125 | 1.2% |  |  |  |  |
| Partial use |  |  | 2 (2.0) | 71 (3.3) | - | - |
| Consistent use |  |  | 22 (21.8) | 298 (14.0) | **1.7 (1.0-2.7)** | **1.7 (1.0-2.8)^f^** |
| Paternal smoking^e^ | 98/1,874 | 12.4% | 32 (32.7) | 540 (28.8) | 1.2 (0.8-1.8) | 1.2 (0.8-1.8)^g^ |

BMI = body mass index. Partial use was defined as folic acid supplement use, alcohol use, or smoking at any point during the time period. Consistent use was defined as folic acid supplement use, alcohol use, or smoking during the entire time period. ^a^ Some cases and controls were excluded in the specific analysis because of missing data on the determinant and/or the confounder(s). ^b^ Cases and controls born after 1994 were included only (N=87/1,716 in total). ^c^ Advised period: 4 weeks before pregnancy through week 8 after conception. ^d^ Etiologically relevant period: week 3 through week 10 after conception.

^e^ During the etiologically relevant period: week 3 through week 10 after conception. ^f^ Adjusted for the birth year categories. ^g^ None of the potential confounders proved to be true confounders.

Supplemental Table 2. Associations between maternal chronic illnesses and the VACTERL association in offspring when maternal medication use was also taken into account to identify mothers with a chronic illness.

|  | Total  cases/controls^a^  (N=142/2,135) | Missing  Data | Cases and controls with potential risk factor  N (%) | | Crude OR  (95% CI) | Adjusted OR  (95% CI) |
| --- | --- | --- | --- | --- | --- | --- |
| Pregestational diabetes | 121/2,126 | 1.3% | 1 (0.8) | 9 (0.4) | - | - |
| CLOPD | 116/1,995 | 7.3% | 9 (7.8) | 59 (3.0) | **2.8 (1.3-5.7)** | **2.1 (1.0-4.4)^b^** |

CLOPD = chronic lower obstructive pulmonary disorders, OR = odds ratio, CI = confidence interval. ORs were estimated if ≥3 cases were exposed. ^a^ Some cases and controls were excluded in the specific analysis because of missing data on the determinant and/or the confounder(s). ^b^ Adjusted for the pregestational BMI and birth year categories.

Supplemental Table 3. Associations between folic acid supplement use and the VACTERL association in offspring by including mothers who reported folic acid use, but did not specify the exact period of folic acid supplement use as: consistent users (1), partial users (2), and non-users (3) in three consecutive simulated analyses.

|  | Total  cases/controls^a^  (N=112/1,716) | Missing  data | Cases and controls with  potential risk factor N (%) | | Crude OR  (95% CI) | Adjusted OR  (95% CI) |
| --- | --- | --- | --- | --- | --- | --- |
| Simulation 1: women without time period specification included as consistent users | | | | | | |
| Advised period^b^ | 104/1,706 | 1.0% |  |  |  |  |
| Partial use |  |  | 36 (34.6) | 490 (28.7) | 1.2 (0.7-2.0) | 0.8 (0.4-1.3)^d^ |
| Consistent use |  |  | 45 (43.3) | 846 (49.6) | 0.9 (0.5-1.4) | **0.5 (0.3-0.9)**^d^ |
| Etiological relevant period^c^ | 104/1,706 | 1.0% |  |  |  |  |
| Partial use |  |  | 20 (19.2) | 251 (14.7) | 1.4 (0.8-2.7) | 0.9 (0.5-1.7)^d^ |
| Consistent use |  |  | 59 (56.7) | 1,002 (58.7) | 1.1 (0.7-1.7) | 0.7 (0.4-1.1)^d^ |
| Simulation 2: women without time period specification included as partial users | | | | | | |
| Advised period^b^ | 104/1,706 | 1.0% |  |  |  |  |
| Partial use |  |  | 54 (51.9) | 907 (53.2) | 1.0 (0.6-1.6) | 0.6 (0.4-1.1)^d^ |
| Consistent use |  |  | 27 (26.0) | 429 (25.1) | 1.0 (0.6-1.8) | **0.5 (0.3-1.0)**^d^ |
| Etiological relevant period^c^ | 104/1,706 | 1.0% |  |  |  |  |
| Partial use |  |  | 33 (31.7) | 554 (32.5) | 1.1 (0.6-1.8) | 0.8 (0.4-1.3)^d^ |
| Consistent use |  |  | 46 (44.2) | 699 (41.0) | 1.2 (0.7-2.0) | 0.7 (0.4-1.2)^d^ |
| Simulation 3: women without time period specification included as non-users | | | | | | |
| Advised period^b^ | 104/1,706 | 1.0% |  |  |  |  |
| Partial use |  |  | 36 (34.6) | 490 (28.7) | 1.4 (0.9-2.2) | 1.1 (0.7-1.8)^d^ |
| Consistent use |  |  | 27 (26.0) | 429 (25.1) | 1.2 (0.7-2.0) | 0.8 (0.5-1.4)^d^ |
| Etiological relevant period^c^ | 104/1,706 | 1.0% |  |  |  |  |
| Partial use |  |  | 20 (19.2) | 251 (14.7) | 1.6 (0.9-2.8) | 1.1 (0.6-2.0)^d^ |
| Consistent use |  |  | 46 (44.2) | 699 (41.0) | 1.3 (0.8-2.0) | 0.9 (0.5-1.4)^d^ |

Partial use was defined as folic acid supplement use at any point during the time period. Consistent use was defined as folic acid supplement use during the entire time period.

^a^ Some cases and controls were excluded in the specific analysis because of missing data on the determinant and/or the confounder(s).

^b^ Advised period: 4 weeks before pregnancy through week 8 after conception. ^c^ Etiological relevant period: week 3 through week 10 after conception.

^d^ Adjusted for the birth year categories.
